# A Holistic View of the Interactions between Electron-Deficient Systems: Clustering of Beryllium and Magnesium Hydrides and Halides

**DOI:** 10.3390/molecules28227507

**Published:** 2023-11-09

**Authors:** Otilia Mó, M. Merced Montero-Campillo, Manuel Yáñez, Ibon Alkorta, José Elguero

**Affiliations:** 1 Departamento de Química, Módulo 13, Facultad de Ciencias, and Institute of Advanced Chemical Sciences (IAdChem), Universidad Autónoma de Madrid, Campus de Excelencia UAM-CSIC, Cantoblanco, 28049 Madrid, Spain; otilia.mo@uam.es (O.M.); manuel.yanez@uam.es (M.Y.); 2 Instituto de Química Médica, IQM-CSIC, Juan de la Cierva, 3, 28006 Madrid, Spain; iqmbe17@iqm.csic.es

**Keywords:** Be and Mg hydrides and halides, clusters, bonding, rehybridization, stability trends, multicenter bond

## Abstract

In the search for common bonding patterns in pure and mixed clusters of beryllium and magnesium derivatives, the most stable dimers and trimers involving BeX_2_ and MgX_2_ (X = H, F, Cl) have been studied in the gas phase using B3LYP and M06-2X DFT methods and the G4 ab initio composite procedure. To obtain some insight into their structure, stability, and bonding characteristics, we have used two different energy decomposition formalisms, namely MBIE and LMO-EDA, in parallel with the analysis of the electron density with the help of QTAIM, ELF, NCIPLOT, and AdNDP approaches. Some interesting differences are already observed in the dimers, where the stability sequence observed for the hydrides differs entirely from that of the fluorides and chlorides. Trimers also show some peculiarities associated with the presence of compact trigonal cyclic structures that compete in stability with the more conventional hexagonal and linear forms. As observed for dimers, the stability of the trimers changes significantly from hydrides to fluorides or chlorides. Although some of these clusters were previously explored in the literature, the novelty of this work is to provide a holistic approach to the entire series of compounds by using chemical bonding tools, allowing us to understand the stability trends in detail and providing insights for a significant number of new, unexplored structures.

## 1. Introduction

Electron-deficient compounds very often exhibit peculiar bonding characteristics. The most paradigmatic example is diborane, in which the two BH_3_ monomers share two hydrogen atoms. As a result of the formation of this quite singular bond, three centers share a pair of electrons as in any typical two-center covalent bond [1,2]. These bonding arrangements, usually named three-center two-electron (3c-2e) bonds, are also found in dialane, the corresponding aluminum hydride dimer. On top of these singular bonding patterns, electron-deficient compounds often present elusive aggregates. A typical example is the corresponding aluminum hydride, whose dimer was predicted to be stable by ab initio calculations [3,4] in the late 1980s but would not be characterized experimentally for the first time until the beginning of the XXI century [5]. In contrast to the stability of diborane, diborane halides, namely B_2_F_6_ and B_2_Cl_6_, which were supposedly not stable in the gas phase [6], were very recently found to be weakly bound, as revealed by high-level ab initio calculations. The low stabilization enthalpies are due to the fact that interaction between monomers is mainly dispersion [7]. In the same paper, a systematic study of dimers and trimers involving BX_3_ and AlX_3_ (X = H, F, Cl) in the gas phase showed that besides dispersion, the rehybridization of the electron-deficient element and its ability to reach pentacoordination are key factors to understand their structure, stability, and bonding characteristics. These findings prompted us to explore the behavior of similar dimers and trimers involving electron-deficient elements from group IIA of the periodic table. Accordingly, in the present paper, we present a theoretical examination of dimers and trimers involving BeX_2_ and MgX_2_ (X = H, F, Cl) in the gas phase.

Not surprisingly, BeH_2_ and its dimer have received a lot of attention. The first two studies we are aware of were on Be_2_H_4_ and Be_3_H_6_ [8] at the Hartree–Fock level, with an estimation of electron correlation effects, and on Be_2_H_4_ and Mg_2_H_4_ using non-empirical approaches, the latter one including also the mixed BeMgH_4_ dimer [9]. A rather complete survey of the dimers, trimers, and tetramers of BeH_2_ and MgH_2_ using high-level ab initio calculations was published in 2005 [10]. More recently, an analysis of the properties of Be_2_H_4_ and other beryllium hydride oligomers and their spectral characteristics have been reported [11], but the Be_3_H_6_ trimer was not included in this survey [11]. In 2011, a study of the interactions of BeH_2_ and Mg_2_H_4_ with H_2_ included information on the structure and energetics of these two clusters, revealing their ability for hydrogen storage purposes [12]. The Raman spectrum of Be_2_H_4_ (D_2*h*_) was predicted [13] using a new code from variational configuration interaction theory to allow the calculation of such spectra in a pure ab initio fashion [14]. The magnesium hydride dimer was characterized by matrix infrared spectroscopy, which led to the conclusion that Mg_2_H_4_ is a dibridged molecule analogous to dialane [15]. Some attention was also paid to polymers of BeH_2_ [16,17]. The information is scarcer for the BeF_2_, MgF_2,_ BeCl_2_, and MgCl_2_ dimers. It can be reduced to Hartree–Fock calculations on Be_2_F_4_, Mg_2_F_4_, and BeF_2_MgF_2_ clusters [18], calculations based on polarized models for MgF_2_ and MgCl_2_ [19], SCF calculations on the dimers of BeF_2_, BeCl_2_, MgF_2_, and MgCl_2_ [20], and electron diffraction experiments on the MgCl_2_ dimer [21]. For the specific case of the BeCl_2_ dimer, gas-phase electron diffraction (GED) and mass spectrometric (MS) experiments allowed us to obtain its structure assuming a D*_2h_* symmetry, and its force constants and frequencies were also estimated [22]. Some complexes involving Be_2_Cl_4_ and phosphorus-containing compounds have been very recently synthesized and structurally characterized, evidencing the Lewis acid behavior of beryllium in the dimer [23]. However, we are not aware of theoretical studies on trimers involving BeX_2_ (X = H, F, Cl) derivatives, with the only exception of the BeH_2_ trimer from [8,10]. The magnesium fluorides and chlorides have instead received most of the attention. To our knowledge, the first survey on the structure and energetics of (MgF_2_)_n_ clusters up to n = 24 was published in 1995 [24]. Years later, the structure of MgF_2_ clusters, from dimers to decamers, was studied [25] using a stochastic optimization technique, namely a genetic algorithm (GA) in association with density functional theory. The structures of Mg_3_Cl_6_ clusters, optimized at the MP2/6-311G* level, were reported in a paper that presents a systematic study of 168 isomers of (MgCl_2_)_n_, where n = 1–4 [26]. It is worth noting that, with the exception of the BeMgH_4_ [9] and BeMgF_4_ [18] dimers, there is no information on mixed dimers or trimers involving BeX_2_ and MgX_2_ (X = H, F, Cl) compounds. The aim of this paper is to present a systematic theoretical study not only of the corresponding homodimers and homotrimers but also of the mixed dimers and trimers. Although, as indicated above, previous studies have been reported for some of these systems, most of them were focused exclusively on structures and energetics. In our study, we will focus our attention on the bonding characteristics of these clusters to precisely explain their structures and relative stabilities. We wonder what changes are observed in the bonding of the dimers when the two metals involved are not identical and how the nature of the substituent may affect bonding and relative stability trends. A similar scenario arises when dealing with trimers. What are the effects on bonding and stability when the dimer interacts with a monomer of the same nature as those forming the dimer? What occurs if the third component is a monomer different from those forming the dimer? How do these effects depend on the nature of the substituent? These are typical questions we will try to answer in this publication. 

## 2. Results and Discussion

Since we are using high-level ab initio calculations and DFT approaches, we first compare the stabilization energies obtained with both methods. The correlation between the G4 and M06-2X values is very good (see Figure S1 of the Supplementary Materials), whereas the correlation with B3LYP functional results is worse (see Figure S2 of the Supplementary Materials). It should be noted that when dispersion contributions are included in our B3LYP calculations using the D3BJ empirical dispersion term, the total stabilization enthalpy becomes more negative from 8 to 20 kJ·mol^−1^, improving the agreement with the G4 outcomes. However, the effects of including dispersion on the geometry of the cluster are negligibly small, to the point that if the B3LYP + D3BJ geometry is used in the standard G4 formalism (that does not include dispersion corrections in the geometry optimization procedure), the changes in the final G4 energy are typically smaller than 0.4 kJ·mol^−1^. Hence, the conclusion is that the G4 formalism is reliable for investigating this type of complexes, in contrast to the results obtained when dealing with B and/or Al derivatives, where the dispersion effects in some of the complexes are a key factor for a proper description [7]. Additionally, in a few cases that will be commented on later, the G4 formalism predicts stationary points that, if obtained, including dispersion corrections, would collapse to the global minimum. In view of this, for the sake of simplicity, we decided to make our discussion using the M06-2X calculated values, knowing that the general conclusions are fully in line with the G4 values. All investigated clusters are closed-shell systems with stable wavefunctions. 

### 2.1. Dimers

**Figure 1:**
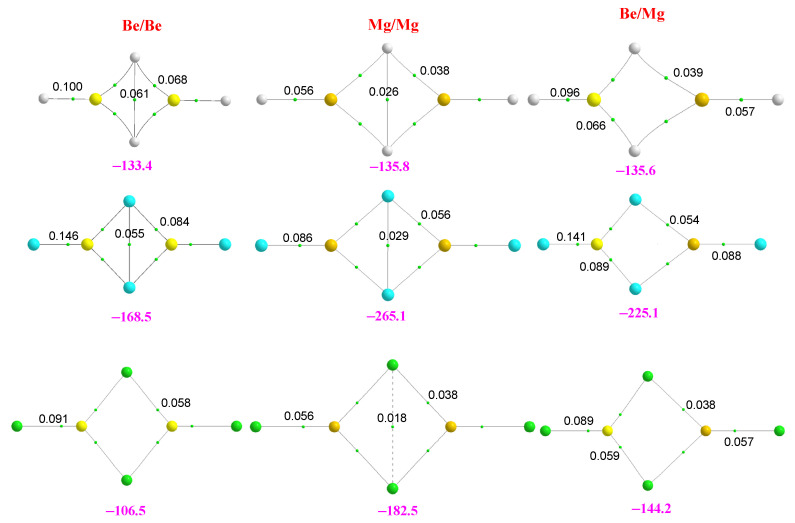
Molecular graphs of the homo and heterodimers involving BeX_2_ and MgX_2_ (X = H, F, Cl) monomers. The electron densities at the bond critical points (BCPs) are in a.u. The numbers in magenta are the stabilization enthalpies in kJ·mol^−1^. Atomic colors code: Be (yellow), Mg (orange), H (white), F (blue), Cl (green).

Standard graphical programs visualize the clusters under investigation with bonding connections based on default internuclear distances, which may lead to an ambiguous description of the systems depending on the software used. Therefore, instead, we will use the molecular graphs of the clusters in all figures in such a way that we can simultaneously have information on the shape and networking of bonds stabilizing the clusters. Following this univocal criterion, we summarized our results for the dimers in Figure 1.

Quite unexpectedly, we found that the stabilization enthalpies of the hydrides are practically equal for the three dimers, both the homodimers containing Be and Mg and the heterodimer. This is not the case, however, for the corresponding halides, in which the magnesium homodimers are clearly more stable than the beryllium homodimers and the heterodimers. It can also be observed that the largest stabilization enthalpies are those of the fluorides, being those of the chlorides not very different from those of the hydrides. Another very interesting result is that, independently of the nature of the substituent, the stabilization enthalpy of the heterodimers is very close (for the chlorides equal) to the average of the stabilization enthalpies of the corresponding homodimers. The latter observation was already suggested by Kirillov et al. for the dimer hydrides [9].

The rationalization of these findings required a detailed analysis of the bonding of these clusters. The first question we want to address is the nature of the bonds in these clusters and how they depend on the substituents. To quantify these two questions, we have carried out an LMO-EDA analysis whose results are shown in Table S1 of the Supplementary Materials. The first conspicuous fact is that the ionic character of the bonds is significant already in the hydrides, where the percentages of electrostatic and exchange components are similar, the former being slightly higher than the latter. For fluorides, the electrostatic component becomes dominant, while for chlorides, the situation is again similar to that of hydrides. This finding is fully consistent with the values of the Laplacian of the electron density, which is, in all cases, positive. However, whereas for the hydrides, the values are rather small and slightly larger for the chlorides, for fluorides, the values are from 3 to 6 times larger. The same trends are observed for the corresponding energy density values (see Figure S3 of the Supplementary Materials). The larger electrostatic weight in the fluorides is consistent with the much larger natural positive charge of Be and Mg when bonded to F and Cl (+1.7 and +1.8) with respect to the ones in the hydrides (+1.1 and +1.2). Even though the charges are similar for fluorides and chlorides, the weight of the electrostatic component is smaller in the latter due to the larger size of the substituent, which results in longer bond lengths. Another important difference between hydrides and halides, which is related to the higher covalent character of the bonds of the former, is that the three hydride dimers are stabilized by the formation of 3c-2e bonds. These bonds are not present in the halides due to the dominant electrostatic character of the interaction, as illustrated by the AdNDP analysis shown in Table S2 of the Supplementary Materials. 

**Figure 2:**
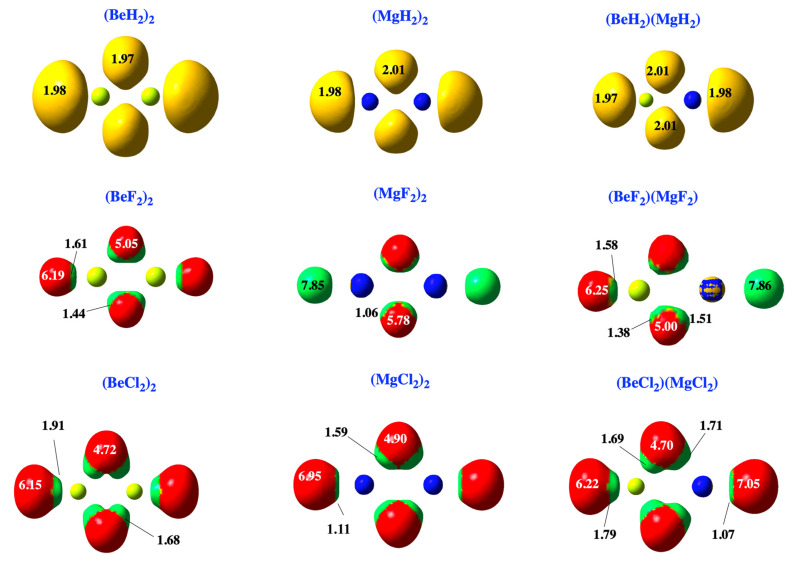
ELF (0.8) disynaptic and trisynaptic basins for the hydride, fluoride, and chloride homodimers and heterodimers. Basins involving H atoms are colored in yellow, and core basins involving Mg appear in dark blue. For the halides, disynaptic basins between the metal atom and fluorine atom appear in green, while lone pairs belonging to halogen atoms are colored in red. Populations are shown in atomic units (e).

This result is consistent with the ELF plots (see Figure 2). It can be seen that the hydrides present trisynaptic Be-H-Be, Mg-H-Mg, and Be-H-Mg basins with a population of two electrons. In contrast, only disynaptic ones are found for fluorides, reflecting the polarization of the lone pairs of fluorine by the positive charge of the metal. The same is found for chlorides, though the polarization of the chlorine lone pairs is clearly larger, as well as the corresponding populations. 

It is also worth noting that, as shown in Table 1, the dispersion contributions in these systems are not negligible, accounting for 6 to 9% of the total stabilization energy, explaining why the B3LYP+D3BJ energies are 9 to 20 kJ·mol^−1^ lower than the B3LYP ones. 

The stability trends within each dimer family still remain to be explained. For this purpose, it is very useful to carry out a MBIE energy decomposition analysis, which is shown in Table 1. 

Starting from the hydrides, it is clear that although the stabilization energies E_total_ are practically equal for the three dimers, the stabilizing two-center energy contributions, Δ^2^*E*(*AB*), are not, being the largest one that of the BeBeH_4_ binary complex. In contrast, the monomer distortion *E_R_* in the BeBeH_4_ cluster is almost twice that of MgMgH_4_. Indeed, the largest contribution to this term comes from the rehybridization undergone by the metal from *sp* to *sp*^n^ (n ≈ 2), but this energy cost is much larger for BeH_2_ than for MgH_2_ (80 vs. 50 kJ·mol^−1^) [27]. Therefore, although the attractive two-center term is larger for Be_2_H_4_, the larger monomer deformation energy compensates for the difference. The rehybridization cost also explains the trends for the halides. In this case, the attractive two-center contributions are rather similar, reflecting the electrostatic character of the interaction, but again, the rehybridization cost is much higher for BeF_2_ than for MgF_2_. Accordingly, the stabilization energy of the MgMgF_4_ cluster is larger than that of the BeBeF_4_ analog. Similar arguments explain the trends for the chlorides. 

Finally, the stabilization enthalpy of the mixed dimers is very close to the mean of the enthalpies of the two homodimers, reflecting that the intrinsic characteristics of each monomer are essentially conserved in the dimer. This is corroborated by the different analyses. The AIM shows that the electron density at the BCP of the BeX_2_ subunit practically does not change when going from the BeX_2_-BeX_2_ dimer to the BeX_2_-MgX_2_ one, and the same can be said as far as the MgX_2_ subunit is concerned. The same conclusion is reached when looking at the characteristics and populations of the ELF basins, the components of the MBIE decomposition analysis, or the Wiberg bond indexes [28] (see Table S3 of the Supplementary Materials). 

### 2.2. Homotrimers

**Figure 3:**
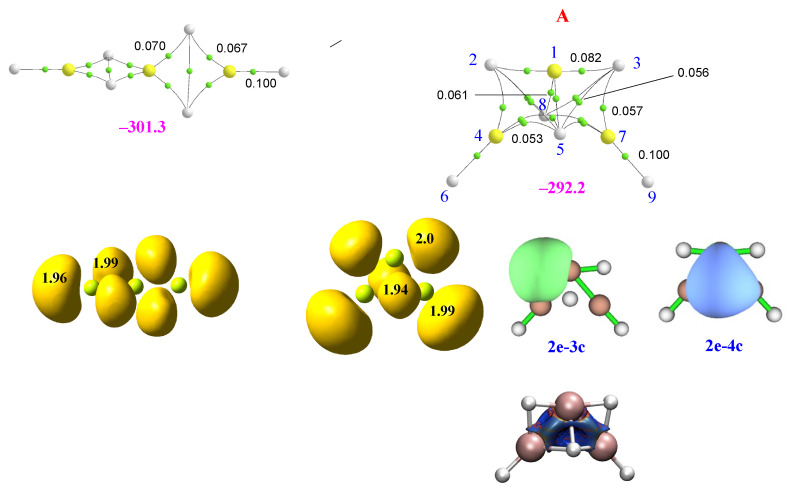
Molecular graphs and ELF plots of the homotrimers involving BeH_2_. The electron densities at the bond critical points (BCPs) are in a.u. The numbers in magenta are the stabilization enthalpies in kJ·mol^−1^. The second row shows the ELF (0.8) trisynaptic basins and their populations, as well as the 3c-2e and 4c-2e orbitals involved in the bonding of cycle **A** obtained by means of the AdNPD approach. In the third row, the 3D representation of non-covalent interactions obtained with NCIPLOT (*s* = 0.3); color code: red (strongly repulsive), green (weakly attractive and weakly repulsive), blue (strongly attractive). A less stable hexagonal trimer (not shown in the figure) is also a local minimum of the potential energy surface.

The two most stable trimers of BeH_2_ are shown in the first row of Figure 3. The most stable one corresponds to a linear aggregate, whereas the second is a cyclic structure labeled **A**. 

Both ternary clusters can be seen as the result of the attachment of a BeH_2_ monomer to the Be_2_H_4_ dimer, in the first case along the Be-Be axis and in the second case perpendicular to it. Accordingly, they present a very different bonding pattern, though the energy gap between them is rather small (9 kJ·mol^−1^). This is the result, as we will discuss below, of subtle differences between the different energy components. If the stabilization enthalpy of the dimer (see Figure 1) is compared with those of the two trimers (see Figure 3), it is evident that the trimerization is followed by some kind of cooperativity since both trimers’ stabilization energies are more than twice the stabilization energy of the dimer. As shown in Figure 3, the central Be atom is tetracoordinated, and according to both the AdNDP and ELF analyses, it is involved in two Be-H-Be bonds with each of the terminal Be atoms. Note, however, that the electron densities at the corresponding BCPs are greater than in the dimer, indicating stronger bonding interactions. Consistently, the ELF analysis finds trisynaptic basins in the trimer, similar to those in the dimer (see second row of Figure 3). These trisynaptic basins also have a population very close to 2 e but within a smaller volume (122 vs. 129 au^3^), whereas the volume of the disynaptic Be-H basins of both terminal groups remains unchanged. This contraction of the trisynaptic basins in the trimer is reflected in a shortening (0.03 Å) of the distance between the central Be atom and the terminal ones with respect to the dimer, reflecting a reinforcement of the interaction. This is also coherent with the MBIE partition energy shown in Table 2 compared to that in Table 1. The distortion energy of the terminal Be atoms are equal in the dimer and the trimer, whereas that of the central Be atom becomes about 40 kJ·mol^−1^ greater as beryllium undergoes a change of hybridization from sp^2^ to sp^3^ to become tetracoordinated. Consistently, the two-center contributions in the trimer are almost identical to those in the dimer, but the additional three-center term leads to its enhanced stabilization. 

The bonding pattern of the cyclic minimum **A** is rather different. In this case, the three Be bonds are tetracoordinated, and non-covalent H···H interactions between the negatively charged hydrogens are also detected. The presence of these interactions implies a certain increase in the dispersion contributions to the stabilization energy, which in the cyclic trimer are significantly larger (−198 kJ·mol^−1^) than in the linear cluster (−148 kJ·mol^−1^). NCIPLOT shows (see third row of Figure 3) that, due to these non-covalent interactions, there is a strongly attractive and quite homogeneous interstitial density between the metals and the hydrogens, in line with the concentration of BCPs found in this area when using AIM. It can also be observed that the dimer subunit is, in this case, significantly distorted, with curved Be-H-Be bond paths, whereas the distortion of the third BeH_2_ subunit is very small, with an almost linear arrangement. This subunit also appears connected to the dimer through the hydrogen atoms of the former. The AdNDP description provides some interesting additional information, showing that the connectivity between 1Be (see numbering in Figure 3) and the dimer subunit takes place through 1Be-2H-4Be and 1Be-3H-7Be 3c-2e bonds and through 1Be-5H-7Be-8H and 1Be-4Be-5H-8H 4c-2e bonds (both kinds depicted in the second row of Figure 3). The ELF description is not strictly identical since all the basins are trisynaptic, though the ones involving 4Be-5(8)H-7Be are more compact (volume 97.5 au^3^) than those involving 1Be-2H-4Be and 1Be-3H-7Be (volume 141.4 au^3^). The MBIE decomposition analysis shows (see Table 2) that the two-center term between the Be atoms of the dimer subunit is smaller in absolute value than in the linear trimer (−202.1 vs. −262.0 kJ·mol^−1^), but the interaction of these two Be atoms with the third Be atom is more than double in cycle **A** (−66.8 vs. −30.9 kJ·mol^−1^), reflecting the formation of the aforementioned 3c-2e bonds. Still, the overall attractive components in **A** are 47 kJ·mol^−1^ above the linear ones. Nevertheless, this difference reduces to only 9 kJ·mol^−1^ in the stabilization energy due to the *E_R_* deformation energies. Indeed, the *E_R_* distortion values of the dimer subunit of cluster **A** are higher than in the same subunits of the linear complex (87 vs. kJ·mol^−1^). In contrast, the *E_R_* value for the 1Be is much smaller (8 kJ·mol^−1^) than that of the central Be atom of the linear trimer (102 kJ·mol^−1^). Accordingly, the overall destabilization energies in the linear isomer are 38 kJ·mol^−1^ greater than in the cyclic one, reducing the gap between their stabilization energies in this amount.

The two main conclusions are that (a) it is not enough to look at the strength of the binding interactions but also at the cost of distortion they entail, and (b) the presence of weak non-covalent interactions indicates that a good description of the geometries of these cyclic clusters requires to account for dispersion, which is not contemplated in the geometry optimization of the standard G4 formalism. 

**Figure 4:**
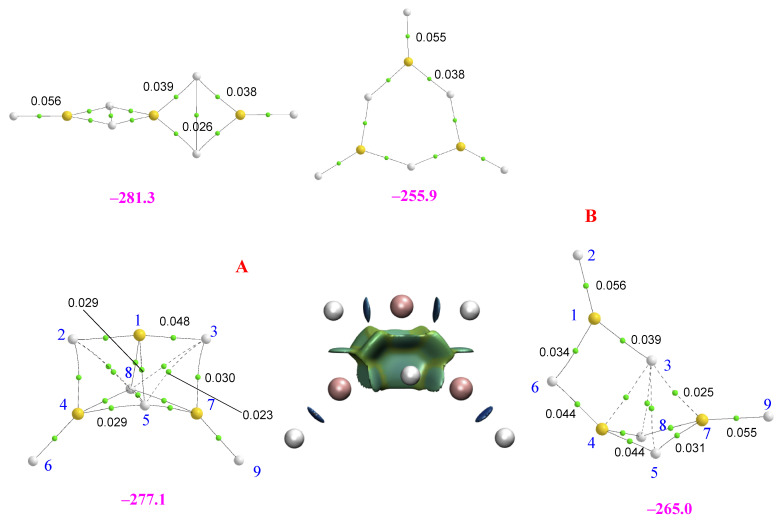
Molecular graphs of the homotrimers involving MgH_2_. The electron densities at the bond critical points (BCPs) are in a.u. The numbers in magenta are the stabilization enthalpies in kJ·mol^−1^. For cluster **A,** the 3D representation of non-covalent interactions obtained with NCIPLOT (*s* = 0.3); color code: red (strongly repulsive), green (weakly attractive and weakly repulsive), blue (strongly attractive), is included.

For the MgH_2_ trimers, the scenario, as illustrated in Figure 4, is a little more complicated, with five (only four shown in the figure) low-energy conformers instead of two. Note that the linear structure is still the global minimum.

From the four cyclic local minima of the potential energy surface, the most stable one is again cycle **A**. This structure is followed in stability by another cyclic structure, **B**, that, as with the previous one, can be seen as the result of the interaction of a MgH_2_ monomer with the MgH_2_-MgH_2_ dimer. Cycles **A** and **B** are distinguished by the *cis* or *trans* arrangement of hydrogens 6 and 9 in the dimer, with the result that in **B,** the 1Mg atom is only tricoordinated. At this point, it should be noted, as mentioned previously, that for the BeH_2_ trimers, only **A** was found to be stable, as any attempt to find **B** led to the linear trimer. The third cyclic isomer in terms of stability is a planar hexagonal structure, though another non-planar conformer (not shown in Figure 4) was found to be also a local minimum, but 7 kJ·mol^−1^ less stable. The bonding analysis discussed above for the BeH_2_ trimers (linear and **A)** can be extended to the MgH_2_ ones, and for similar reasons, again, the linear tautomer is slightly more stable than the trimer **A**. Specifically, the linear MgMgMgH_6_ trimer is 20 kJ·mol^−1^ less stable than its Be-containing analogue. For this structure, in line with what was discussed for the dimers, the Δ^2^*E* attractive interactions and the *E_R_* repulsive ones are smaller in MgMgMgH_6_ than in the BeBeBeH_6_ analog (see Table 2), resulting in a smaller stabilizing enthalpy in the former. A comparison of Figure 3 and Figure 4 shows that the structure of cluster **A** for Mg is less compact than the homologous Be-containing isomer due to the longer interatomic distances (see Table S4 of the Supplementary Materials). Consistently, some of the non-covalent interactions are weaker. NCIPLOT shows that, due to its smaller compactness, the attractive homogeneous interstitial density between the Mg atoms and the hydrogens is less strongly attractive than in the Be analog. Going from cluster **A** to **B**, as expected, the overall attractive contributions (Δ^2^*E* and Δ^3^*E*) decrease by about 6 kJ·mol^−1^, whereas the *E_R_* terms increase by about the same amount, explaining why complex **B** is only 12 kJ·mol^−1^ less stable than conformer **A**. The lower stability of the hexagonal cycle just reflects the decrease in the Δ^2^*E* attractive terms because no 3c-2e bonds are formed in this case, which is only partially compensated by an increase of the Δ^3^*E* contribution due to the hexagonal arrangement of this complex. 

**Figure 5:**
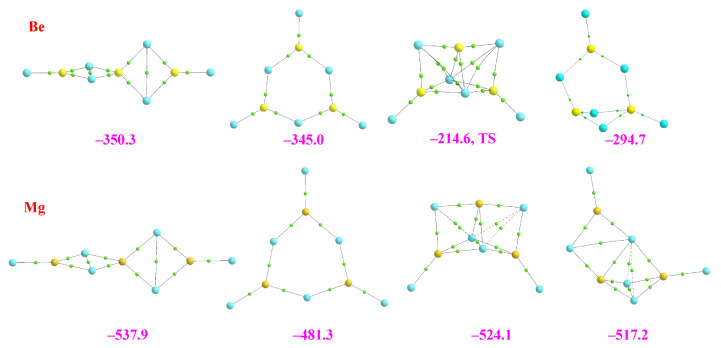
Molecular graphs of the homotrimers involving BeF_2_ and MgF_2_. The numbers in magenta are the stabilization enthalpies in kJ·mol^−1^.

The stability trends of the different conformers change dramatically when moving to the halides, though the linear trimer is still the global minimum. Let us discuss the fluorides shown in Figure 5 in more detail.

For the BeF_2_ trimers, the linear arrangement is the global minimum, followed by the hexagonal cycle, only 5 kJ·mol^−1^ less stable, and by cycle **B,** which is 50 kJ·mol^−1^ further less stable. In this case, cycle **A** is a stationary point with one imaginary frequency. The same clusters are found when Be is replaced by Mg, but in this case, all of them are local minima of the potential energy surface. However, the energy trends are totally different, with cycle **A** being the second most stable after the linear trimer. As in the dimers, the dominant electrostatic character in fluorides renders the linear trimer more stable than the homologous containing BeH_2_ (−350.3 vs. −301.3 kJ·mol^−1^). Conversely, since this dominant electrostatic character prevents the formation of 3c-2e bonds, cycle **A** is found to be significantly less stable than its homologous hydride (−214.6 vs. −277.1 kJ·mol^−1^). Cycle **A** is indeed much less stable than the linear trimer due to its compactness, which results in a close vicinity of F atoms reflected by the repulsive terms in the LMO-EDA results of Table S5. The consequence is that for the fluorides, **A** is a TS that leads to the linear cluster. The MBIE analysis (see Table 2) also shows that the hexagonal cycle is marginally less stable than the linear trimer. This is caused by the very small difference between the attractive (Δ^2^*E* + Δ^3^*E*) and the repulsive (*E_R_*) terms, which is slightly greater (4 kJ·mol^−1^) than in the former, so those species can be considered practically degenerate. The main difference when dealing with the Mg-containing systems is the larger size of Mg and the much longer bonds, which contribute to significantly stabilizing cycles **A** and **B**. These cycles are less compact, closing the gap with respect to the linear global minimum. It should be mentioned that although the MgF_2_ trimers we found coincide with those in the literature [25], this is not the case as far as their stabilization energies are concerned since our G4 and M06-2X calculations both predict a different stability order (see Table S6 of the Supplementary Materials), likely due to the effect of dispersion contributions only included in our calculations.

The structures and stabilities of the homotrimer chlorides are presented in Figure S4 of the Supplementary Materials. As was the case for the hydrides and fluorides, the most stable trimer is the linear one. However, there are some differences with respect to fluorides in what concerns the relative stabilities of the other minima. For BeF_2_ homotrimers, the hexagonal trimer is planar, whereas in the corresponding chloride, it is not. On top of that, the fluoride is 5 kJ·mol^−1^ less stable than the linear conformer, whereas for the chloride, this gap becomes ten times larger. Also, for Be trimers, cycle **A** is found to be a TS and the least stable stationary point, showing once more the significative effect of the repulsion in these compact systems when the substituent is voluminous as Cl. Conversely, for Mg, where these interactions are much weaker due to the much larger interatomic distances (see Table S4 of the Supplementary Materials), cycle **A** is not only a minimum but close in energy to the global minimum. 

### 2.3. Heterotrimers

**Figure 6:**
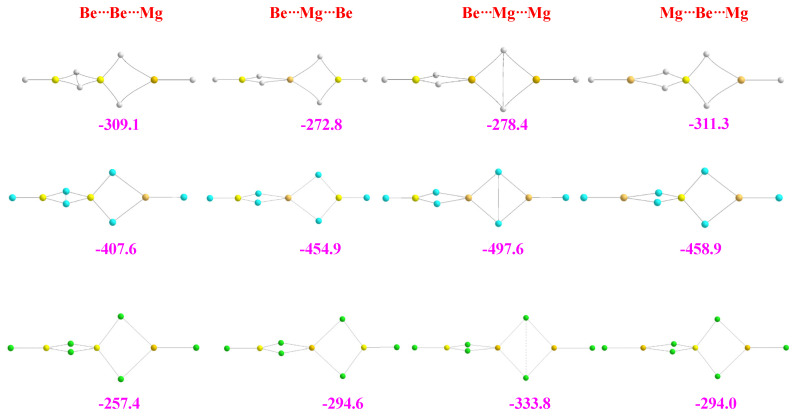
Molecular graphs for the linear heterotrimers, showing their stabilization enthalpies in kJ·mol^−1^.

The conformational richness when dealing with heterotrimers is very high. Starting from the linear clusters, the number multiplies by three on going from homo to heterodimers. This can be easily understood if we remember that a linear trimer can be seen as the result of the interaction of a dimer with a monomer along the axis. Hence, if we take the homodimers X_2_Be-BeX_2_, its interaction can be exclusively with MgX_2_, leading to a unique BeX_2_BeX_2_MgX_2_ arrangement. However, if the interaction involves the heterodimer, X_2_Be-MgX_2_, the interaction can take place with any of the two monomers. The interaction with BeX_2_ will yield the same trimer as before if the attachment takes place on the BeX_2_ or to a new BeX_2_MgX_2_BeX_2_ conformer if this attachment takes place on the MgX_2_ side. If the monomer involved is MgX_2_, two new clusters, MgX_2_BeX_2_MgX_2_ and BeX_2_BeX_2_MgX_2_, would be produced. All these possibilities, with their corresponding stabilization enthalpies, are shown in Figure 6. As expected from our discussion on the dimers, fluorides are significantly more stable than hydrides, whereas chlorides are only slightly more stable or unstable than hydrides, depending on the case. A second conspicuous fact is that this stability depends on the nature of the central atom and that the stabilities observed for the hydrides reverse on going to fluorides and chlorides. Indeed, for the hydrides, the most stable linear clusters of each kind are those in which the central atom is Be, whereas in fluorides and chlorides, the most stable are systematically those in which the central atom is Mg. 

This finding is the result of mainly two features: the distortion energies of the central atom and the three-body contributions to the total energy. As it can be seen in Table S7 of the Supplementary Materials, for the hydrides, *E_R_* is almost twice when the central atom is Be instead of Mg. This effect is more than compensated by the Δ^2^*E* terms, more negative in the first case, but mainly by the Δ^3^*E* term, more than three times larger in the former than in the latter thanks to stronger 3c-2e bonds in H_2_Be-BeH_2_ than in BeH_2_-MgH_2_. When moving to the halides, the Δ^3^*E* contribution is marginal because, in these systems, no 3c-2e bonds are formed. It only remains, as a key factor, the much higher *E_R_* value when the central atom is Be, leading to less stable clusters than those where the central atom is Mg. 

**Figure 7:**
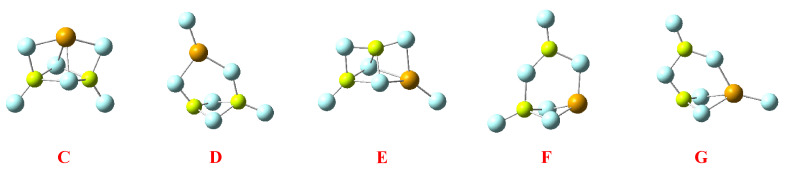
Different kinds of cyclic heterotrimers formed by the interaction of a dimer with a monomer that approaches the former in a direction perpendicular to the dimer axis.

Concerning the cyclic trimers, one option is the formation of hexagonal trimers, also found for the homotrimers. However, these structures were only found to be stable for hydrides and fluorides, but in both cases, more than 50 kJ·mol^−1^ is less stable than the corresponding linear trimers (see Figure S5 of the Supplementary Materials). More interesting are the cycles similar to the clusters **A** and **B** described in the homotrimers section. When dealing with heterotrimers, the different cycles that can be envisaged amount to five instead of two, as shown in Figure 7. 

Cycles **C** and **D** result from the interaction of a homodimer with a different monomer. They differ in the *cis* (**C**) or *trans* (**D**) arrangement of the terminal substituents of the homodimer. On the other hand, cycles **E**, **F,** and **G** arise from the interaction between a heterodimer and a certain monomer. Again, if the substituents of the dimer are *cis*, cycle **E** is formed. If the substituents are *trans,* there are two possibilities, cycles **F** and **G**. The important matter is that in some specific cases, these cycles become the global minimum of the potential energy surface. Let us start with the hydrides. The molecular graphs of these clusters are shown in Table 3, together with their stabilization enthalpies and their relative enthalpies with respect to the corresponding linear trimers in Figure 6. 

The first conspicuous fact is that cycle **G** does not exist because it collapses to the linear global minimum. The second and most important is that some of them are rather stable, to the point that one within each family is predicted to be the global minimum of the potential energy surface (negative relative stabilities in red). This is a result of subtle differences, related again essentially to the distortion energy of the monomers and the Δ^3^*E* terms. In cycle **E** (see Table S8 of the Supplementary Materials), the *E_R_* contributions are smaller than in the linear trimer because the BeH_2_ moiety at the top of the cycle is almost linear. As expected, the three Δ^2^*E* in cycle **E** are negative, whereas in the linear trimer, only those of the central unit with the other two are negative. The overall balance is still favorable to the linear trimer, but this is compensated by a larger Δ^3^*E* contribution, which renders cycle **E** the most stable. For the case of the BeMgMgH_6_ heterotrimers, the situation is slightly different. Now, the *E_R_* contributions are very similar in both cycle **C** and the linear trimer (see Table S8 of the Supplementary Materials) because of a larger distortion of the Be derivative at the top in cycle **C**. Since the Δ^2^*E* contributions are globally similar, even though in the linear trimer, as expected, only two are significantly large, the enhanced stability of cycle **C** comes essentially from the Δ^3^*E* term. Hence, once more, the two key factors are the *E_R_* and the Δ^3^*E* components, and when the former does not contribute significantly, it is only the Δ^3^*E* term that is behind the stability differences. 

When moving to fluorides and chlorides, the scenario changes completely with respect to the relative stability of cycles **C**–**G** (see Table S9 of the Supplementary Materials). Indeed, neither in the fluorides nor in the chlorides do the cyclic trimers compete in stability with the linear ones. This reflects the fact already mentioned in previous sections: the dominant electrostatic character of the interactions avoids the formation of 3c-2e bonds and, accordingly, since these cycles are rather compact structures, the repulsive interactions between the substituents increase significantly, and the Δ^3^*E* contributions become highly unstabilizing (see Table S10 of the Supplementary Materials).

## 3. Computational Details

The first step of our analysis was a screening of the different dimers and trimers that can be possible minima of the corresponding potential energy surface. From them, we chose the most stable form for each of the different isomers, linear or cyclic. It should be mentioned that for the particular cases of BeH_2,_ MgH_2_, MgF_2_, and MgCl_2_ trimers, our most stable structures are the same as those previously reported in the literature [10,24,25,26]. 

The structure and final energies of the clusters under investigation were obtained using both ab initio and density functional theory methods. In the first case, we have used the composite Gaussian-4 (G4) formalism [29]. In this method, the different ab initio calculations are carried out on geometries optimized with the B3LYP DFT approach together with a 6-31G(2df,p) basis set expansion. Final energies are calculated by combining different methods that properly account for the electron correlation effects, namely Møller–Plesset (MPn) perturbation theory up to the fourth order and CCSD(T) coupled-cluster theory. A final correction should be added to this: an estimation of the Hartree–Fock energy limit (HFlimit) together with two high-level empirical corrections. The result is that final energies are accurate up to a CCSD(T,full)/G3LargeXP + HF limit level, with an average absolute deviation [29] of 3.47 kJ·mol^−1^. 

For our DFT calculations, we have chosen the B3LYP method, which is the one used in the G4 formalism for the geometry optimizations. To specifically check whether dispersion effects are significant for the systems under investigation, we have also added to the B3LYP method the D3BJ empirical dispersion term proposed by Grimme, including the Becke–Johnson damping correction [30]. The second DFT method chosen was the M06-2X, as this functional also provides much better quality descriptions of dispersion-dominated systems than standard functionals [31]. Moreover, M06-2X yields values that correlate very well with the MP2 ones, in particular when an extended basis set is used [32,33,34,35]. Very recently, we have found it to provide results in good agreement with G4 calculations when dealing with clusters involving electron-deficient systems [7]. For all these DFT calculations, a rather flexible aug-cc-pVTZ basis set has been used. 

In particular, when dealing with trimers, a good understanding of the nature of bonds in this kind of cluster requires knowing the contribution of one-, two- and three-body terms, as well as the weight of the electrostatic, exchange, and dispersion contributions to the total binding energy.

For the first goal, we have used the many-body interaction energy (MBIE) formalism [36,37], which, for a ternary complex, allows the decomposition of the total binding energy ∆*E* (Equation (1)) into one- (Equation (2)), two- (Equation (3)), and three-body interactions (Equation (4)), as follows:

(1) $$∆E=E\left(ABC\right)-\sum_{i=A}^{C}E_{m}\left(i\right)=\sum_{i=A}^{C}E_{R}\left(i\right)+\sum_{i=A}^{B}\sum_{j>i}^{C}{∆}^{2}E\left(i,j\right)+{∆}^{3}E(ABC)$$

(2) $$E_{R}\left(i\right)=E\left(i\right)-E_{m}\left(i\right)$$

(3) $${∆}^{2}E\left(ij\right)=E\left(ij\right)-\left[E\left(i\right)-E(j)\right]$$

(4) $${∆}^{3}E\left(ABC\right)=E\left(ABC\right)-\left[E\left(A\right)+E\left(B\right)+E\left(C\right)\right]-{[∆}^{2}E\left(AB\right)+{∆}^{2}E\left(AC\right)+{∆}^{2}E\left(BC\right)]$$

The value of *E_R_*(*i*), which measures the energy associated with the monomer distortion when it is part of the trimer, is the difference between *E_m_*(*i*), the energy of the i-monomer in its equilibrium geometry, and *E*(*i*), the energy of the i-monomer within the geometry of the ABC complex. Δ^2^*E*(*ij*) and Δ^3^*E*(*ABC*) are the two- and three-body interaction energies computed at the corresponding geometries in the complex. For the second objective, we have employed the LMO-EDA [38] decomposition analysis based on the generalized Kohn–Sham (GKS) and localized molecular orbitals, which permits us to write the total interaction energy as the sum of electrostatic, exchange, repulsion, polarization, and dispersion contributions (Equation (5)).

E_int_ = E_elec_ + E_exc_ + E_rep_ + E_pol_ + E_disp_ (5)

It should be mentioned that the distortion energy present in the MBIE analysis is not included in the LMO-EDA energy decomposition procedure. The LMO-EDA calculations were carried out by using the GAMESS code (version 2012-R1) [39]. 

To detect the new bonds that stabilize each cluster and to have reliable information on their nature and strength, we have carried out a topological analysis of the molecular electron density, ρ(r), by means of the Atoms in Molecules (AIM) approach [40], using the AIMAll (Version 19.10.12) code [41]. In this way, we could locate its first-order saddle points, the so-called bond critical points (BCPs), and obtain the corresponding molecular paths that provide information on the bonds stabilizing the system. In fact, since an inspection of the cluster structures in terms of interatomic distances and angles does not necessarily lead to knowing which bonds have been formed, the discussion of the cluster structures will be carried out using the corresponding molecular graphs. In all cases, the AIM analysis was followed by NBO (Natural Bond Orbitals) [42] calculations to complement the bonding information, in particular on what concerns the formation of (3c-2e) bonds. These calculations have been carried out with the NBO 5.G code [43]. In this respect, another useful technique to detect the existence of multicenter bonds is the AdNDP analysis [44], which was used for dimers and trimers. For those systems in which non-covalent interactions might be present, the information provided by the AIM method is nicely complemented by the NCIPLOT approach [45], which allows finding regions of low reduced density gradient (*s*) and low-density values typically associated with these non-covalent interactions. 

Further information on the bonding of these clusters may be obtained by using the Electron Localization Function (ELF) formalism [46], which permits the location of the areas in which the electrons of the system are distributed in monosynaptic and disynaptic (or polysynaptic) basins. These regions are characterized by a low value of the excess local kinetic energy, thus identifying highly localized electrons. Monosynaptic basins are typically associated with core and electron lone pairs, whereas disynaptic (or polysynaptic) basins are associated with two-center (or more than two) bonding interactions. 

## 4. Conclusions

Considering the unpaired electron available for bonding in H and halogens, one might expect that clustering between pure and mixed beryllium and magnesium clusters could follow relatively similar stabilities and bonding patterns for these substituents. However, a global picture of the dimers and trimers of this family offers a much more complex panorama. This work has dealt with the reasons behind the observed trends.

We have seen that magnesium homodimers are significantly more stable than beryllium homodimers and heterodimers and that the largest stabilization enthalpies are those of the fluorides, far from hydrides and chlorides, which are more alike. Thanks to LMO-EDA and NBO/AdNDP, we have shown that the electrostatic component is, as expected, much larger in halides than in hydrides, but chlorine has a lower contribution because of its larger size and cannot form multicenter two-electron bonds as hydrides do. The MBIE analysis helped to reveal the importance of the deformation (rehybridization) energy cost, which is much larger for beryllium than for magnesium.

Regarding the hydride homotrimers, we have understood why cyclic structures such as cyclic **A** can compete in stability with the linear structure. As the MBIE showed, in line with observations through topological tools, not only the strength of binding interactions must be taken into account but also the distortion involved to form the structures, together with the presence of non-covalent interactions in cycle **A** that have to be properly included in the theoretical treatment. Instead, beryllium fluoride clusters prefer the linear conformation, avoiding the compactness of cyclic structures, whereas magnesium, with larger bonds, presents cyclic structures **A** and **B** closer in energy to the linear one. 

Finally, we have also paid attention to hydride heterotrimers, locating linear and several cyclic structures, where the many-body interactions allowed us to explain the preferences for central Be or Mg atoms in the linear structures. MBIE can also rationalize the subtle differences in the cyclic cases and explain why, for the BeBeMgH_6_ and BeMgMgH_6_ hydrides, the compact cycles, as **C** and **E**, become the global minima, respectively. However, halogen heterotrimers cannot compete in their cyclic forms with the linear ones in the absence of multicenter two-electron bonds and the predominance of three-body unstabilizing terms. As a final comment, we would like to point out that the study of the interactions between the BeCl_2_ dimers with phosphorus-containing compounds reported in ref. [23] suggests that the clusters investigated here may exhibit a rather interesting reactivity, whose study could be a good benchmark to analyze it in terms of HOMO-LUMO interactions, as proposed in a very recent publication [47]. 

## Figures and Tables

**Table 1:** MBIE analysis of the binary complexes formed by BeX_2_ and MgX_2_ (X = H, F, Cl). All values in kJ·mol^−1^.

Binary Complex	*E_R_*(*A*)	*E_R_*(*B*)	Δ^2^*E*(*AB*)	*E_total_*
*Hydrides*				
BeBeH_4_	58.7	58.7	−261.4	−144.1
MgMgH_4_	36.3	36.3	−216.6	−143.9
BeMgH_4_	66.3	31.6	−243.2	−45.2
*Fluorides*				
BeBeF_4_	84.1	84.1	−340.8	−172.6
MgMgF_4_	42.3	42.3	−353.1	−268.5
BeMgF_4_	85.8	42.6	−357.5	−229.1
*Chlorides*				
BeBeCl_4_	80.5	80.5	−269.8	−108.9
MgMgCl_4_	44.2	44.2	−273.6	−185.3
BeMgCl_4_	88.0	40.0	−274.8	−146.8

**Table 2:** MBIE analysis of the homotrimers formed by BeX_2_ and MgX_2_ (X = H, F). All values in kJ·mol^−1^.

Ternary Complex	*E_R_*(*A*)	*E_R_*(*B*)	*E_R_*(*C*)	Δ^2^*E*(*AB*)	Δ^2^*E*(*AC*)	Δ^2^*E*(*BC*)	Δ^3^*E*(*ABC*)	*E_total_*
BeBeBeH_6_ (linear)	59.2	102.6	59.2	−262.0	7.8	−262.0	−30.9	−326.2
BeBeBeH_6_ (cyclic, **A**)	87.4	8.0	86.7	−202.1	−115.3	−116.0	−66.8	−318.1
BeBeBeH_6_ (hexagonal)	82.9	82.9	82.9	−140.1	−140.1	−140.1	−57.0	−228.7
MgMgMgH_6_ (linear)	37.2	66.8	37.2	−215.2	0.3	−215.2	−10.4	−299.3
MgMgMgH_6_ (cyclic, **A**)	58.4	4.2	58.2	−185.5	−99.6	−99.9	−30.6	−294.8
MgMgMgH_6_ (cyclic, **B**)	28.1	44.5	54.7	−124.6	−54.0	−188.6	−42.0	−281.9
MgMgMgH_6_ (hexagonal)	42.9	42.9	42.9	−100.1	−100.1	−100.1	−99. 8	−271.4
MgMgMgH_6_ (hexagonal, non-planar)	51.6	46.7	28.5	−177.8	−77.2	−67.8	−69.3	−265.3
BeBeBeF_6_ (linear)	84.0	167.3	84.0	−345.2	−2.1	−345.2	−2.0	−359.3
BeBeBeF_6_ (hexagonal)	95.1	95.1	95.1	−175.1	−175.1	−175.1	−114.4	−354.4
BeBeBeF_6_ (cyclic, **A**)	162.2	29.1	162.4	−358.3	−146.2	−146.5	80.1	−217.3
BeBeBeF_6_ (cyclic, **B**)	78.2	124.8	181.4	−139.7	−168.9	−338.5	−40.3	−303.0
MgMgMgF_6_ (linear)	42.4	82.1	42.5	−356.3	0.3	−356.3	1.2	−546.1
MgMgMgF_6_ (cyclic, **A**)	92.3	16.0	92.4	−351.9	−214.4	−214.4	48.2	−531.6
MgMgMgF_6_ (cyclic, **B**)	39.5	56.2	63.1	−250.2	−133.0	−307.4	6.8	−525.0
MgMgMgF_6_ (hexagonal)	47.5	47.6	47.6	−188.5	−188.5	−188.5	−65.8	−488.8

**Table 3:** Molecular graphs of cycles **C**–**G** of BeH_2_BH_2_MgH_2_ and BeH_2_MgH_2_MgH_2_ and their stabilization enthalpies (bold numbers). In blue, their relative stabilities with respect to the corresponding linear trimer are also provided. In red, the two cases in which the cycle is the global minimum. All values in kJ·mol^−1^.

	BeBeMgH_6_		BeMgMgH_6_	
	Molecular graph	Stab. enth./ Rel. Stab.	Molecular graph	Stab. enth./ Rel. Stab.
***Trimers***	**BeBeMg**		**BeMgMg**	
**C**	** 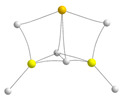 **	**−253.6** **55.5**	** 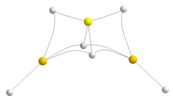 **	**−** **319.5** **−8.2**
**D**	** 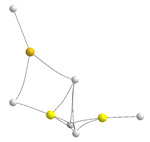 **	**−299.8** **9.3**	** 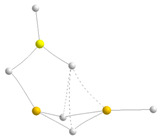 **	**−248.4** **62.9**
**E**	** 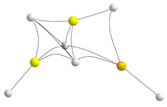 **	**−** **313.7** **−4.6**	** 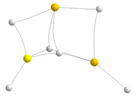 **	**−271.3** **40.0**
**F**	** 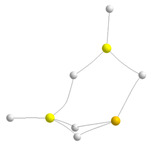 **	**−221.7** **87.4**	** 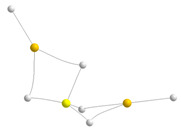 **	**−208.1** **103.2**

## Data Availability

The authors confirm that the data supporting the findings of this study are available within the article and its Supplementary Materials.

## Supplementary Materials

The following supporting information can be downloaded at: https://www.mdpi.com/article/10.3390/molecules28227507/s1. Figure S1. Correlation between G4 and M06-2X interaction enthalpies. Figure S2. Correlation between the interaction enthalpies calculated at the G4 and B3LYP levels of theory. Table S1. LMO-EDA analysis for BeBeX_4_, MgMgX_4_ and BeMgX_4_ (X = H, F, Cl) dimers. Figure S3. Molecular graphs of the homo and heterodimers involving BeX_2_ and MgX_2_ (X = H, F, Cl) monomers, showing the electron density, its Laplacian and the energy density at the bond critical points (BCPs). Table S2. AdNPD orbital list for the BeBeX_4_, MgMgX_4_ and BeMgX_4_ dimers. Table S3. Wiberg bond indexes for Be_2_X_4_, Mg_2_X_4_, BeMgX_4_ (X = H, F, Cl). Table S4. Interatomic distances in the cycles **A** of Be_2_H_4_ and Mg_2_H_4_. Table S5. LMO-EDA analysis for the BeBeBeF_6_ trimers. Figure S4. Bond paths and stabilization enthalpies for the BeCl_2_ and MgCl_2_ homotrimers. Table S6. Relative stabilities for the MgF_2_ trimers obtained by different theoretical approaches. Table S7. MBIE analysis of linear heterotrimer complexes formed by BeX_2_ and MgX_2_ (X = H, F, Cl). Figure S5. Bond paths for the stable hexagonal heterotrimers BeCl_2_ and MgCl_2_ homotrimers, showing their stabilization and relative enthalpies with respect to the corresponding linear heterotrimer in kJ·mol^-1^. Table S8. MBIE analysis of BeBeMgH_6_ and BeMgMgH_6_ heterotrimer complexes. Table S9. Molecular graphs of cycles **C**-**G** of BeX_2_BX_2_MgX_2_ and BeX_2_MgX_2_Mgx_2_ (X = F, Cl) and their stabilization enthalpies. Table S10. MBIE analysis of heterotrimer complexes for fluorides and chlorides.
