# Supplementary material for: A Holistic View of the Interactions between Electron-Deficient Systems: Clustering of Beryllium and Magnesium Hydrides and Halides
Source: Molecules. 2023 Nov 9;28(22):7507. doi: 10.3390/molecules28227507 (PMC10673300; doi:10.3390/molecules28227507)
Supplement: Supplementary file 1 [file molecules-28-07507-s001.zip › molecules-2671038-supplementary.pdf]

A Holistic View on the Interactions between Electron-deficient systems: Clustering of Beryllium and Magnesium Hydrides and Halides.

Otilia Mó[1], M. Merced Montero-Campillo[1*], Manuel Yáñez[1*], Ibon Alkorta[2*], José Elguero[2].

[1] Departamento de Química, Módulo 13, Facultad de Ciencias, and Institute of Advanced Chemical Sciences (IAdChem), Universidad Autónoma de Madrid, Campus de Excelencia UAM-CSIC, Cantoblanco, 28049 Madrid, Spain.

[2] Instituto de Química Médica, IQM-CSIC, Juan de la Cierva, 3. 28006 Madrid, Spain.
mm.montero@uam.es, manuel.yanes@uam.es, ibon@iqm.csic.es

CONTENTS

Figure S1. Correlation between G4 and M06-2X interaction enthalpies.

Figure S2. Correlation between the interaction enthalpies calculated at the G4 and B3LYP levels of theory.

Table S1. LMO-EDA analysis for $BeBeX_4$, $MgMgX_4$ and $BeMgX_4$ (X = H, F, Cl) dimers.

Figure S3. Molecular graphs of the homo and heterodimers involving BeX_2 and MgX_2 (X = H, F, Cl) monomers, showing the electron density, its Laplacian and the energy density at the bond critical points (BCPs).

Table S2. AdNPD orbital list for the $BeBeX_4$, $MgMgX_4$ and $BeMgX_4$ dimers.

Table S3. Wiberg bond indexes for Be_2X_4, Mg_2X_4, $BeMgX_4$ (X = H, F, Cl)

Table S4. Interatomic distances in the cycles **A** of Be_2H_4 and Mg_2H_4.

Table S5. LMO-EDA analysis for the $BeBeBeF_6$ trimers.

Figure S4. Bond paths and stabilization enthalpies for the $BeCl_2$ and $MgCl_2$ homotrimers.

Table S6. Relative stabilities for the MgF_2 trimers obtained by different theoretical approaches.

Table S7. MBIE analysis of linear heterotrimer complexes formed by BeX_2 and MgX_2 (X = H, F, Cl).

Figure S5. Bond paths for the stable hexagonal heterotrimers $BeCl_2$ and $MgCl_2$ homotrimers, showing their stabilization and relative enthalpies with respect to the corresponding linear heterotrimer in kJ·mol^{-1}.

Table S8. MBIE analysis of $BeBeMgH_6$ and $BeMgMgH_6$ heterotrimer complexes.

Table S9. Molecular graphs of cycles **C-G** of $BeX_2BX_2MgX_2$ and $BeX_2MgX_2Mgx_2$ (X = F, Cl) and their stabilization enthalpies

Table S10 . MBIE analysis of heterotrimer complexes for fluorides and chlorides.

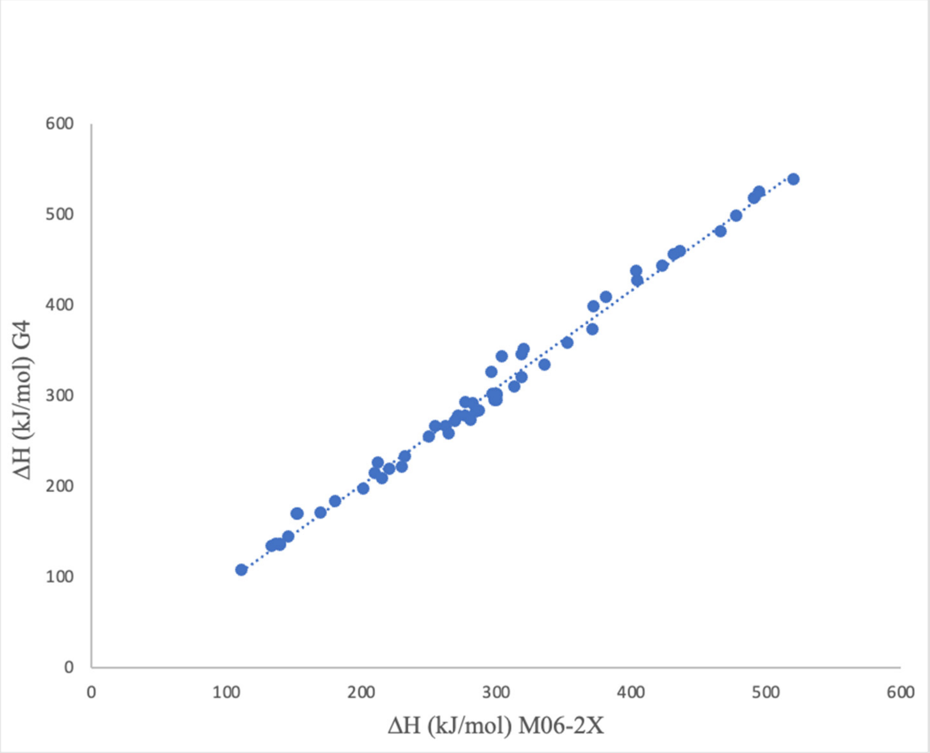

Figure S1. Correlation between the interaction enthalpies calculated at the G4 and M06-2X levels of theory for $BeBeX_4$, $MgMgX_4$, $BeMgX_4$, $BeBeBeX_6$, $MgMgMgX_6$, $BeBeMgX_6$, and $BeMgMgX_6$ (X = H, F, Cl) clusters (ΔH G4 = 1.0735 ΔH M062X - 13.890; R^2 = 0.991).

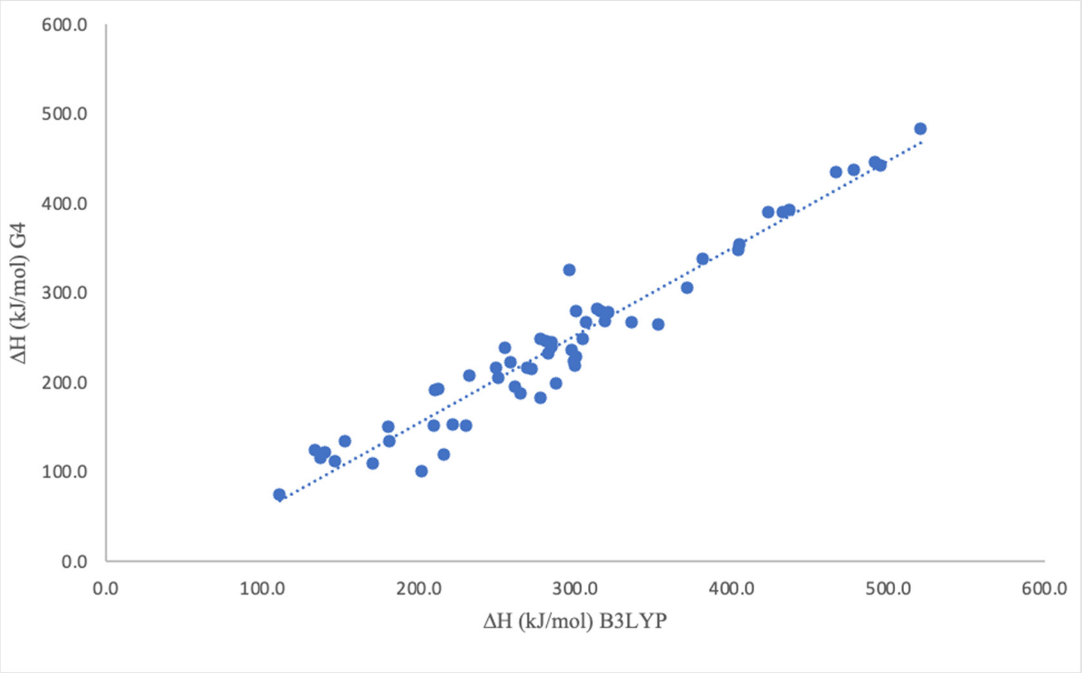

Figure S2. Correlation between the interaction enthalpies calculated at the G4 and B3LYP levels of theory for $BeBeX_4$, $MgMgX_4$, $BeMgX_4$, $BeBeBeX_6$, $MgMgMgX_6$, $BeBeMgX_6$, and $BeMgMgX_6$ (X = H, F, Cl) clusters (ΔH G4 = 0.9777 ΔH B3LYP - 40.213; R^2 = 0.9399).

Table S1. LMO-EDA analysis for the $BeBeX_4$, $MgMgX_4$ and $BeMgX_4$ (X = H, F, Cl) dimers (all values in kJ·mol^{-1})

a) Hydrides

	Energies (kJ·mol^{-1}) of the different components			% of the attractive components to the total E		
	$BeBeH_4$	$MgMgH_4$	$BeMgH_4$	$BeBeH_4$	$MgMgH_4$	$BeMgH_4$
Electrostatic	-425.1	-376.6	-384.4	34.5	39.4	35.4
Exchange	-388.7	-301.5	-342.7	31.5	31.6	31.6
Repulsion	972.6	738.9	841.6			
Polarization	-351.1	-218.0	-293.0	28.5	22.8	27.0
Dispersion	-69.0	-59.4	-64.8	5.6	6.2	6.0
Total E	-261.4	-216.6	-243.4	34.5	39.4	35.4

b) Fluorides

	BeBeF4	MgMgF4	BeMgF4	BeBeF4	MgMgF4	BeMgF4
Electrostatic	-494.2	-498.0	-497.9	42.4	56.9	48.3
Exchange	-263.0	-157.2	-211.6	22.6	18.0	20.5
Repulsion	823.9	521.7	673.2			
Polarization	-311.9	-146.1	-235.8	26.8	16.7	22.9
Dispersion	-95.4	-73.3	-85.6	8.2	8.4	8.3
Total E	-340.7	-352.9	-357.6	42.4	56.9	48.3

c) Chlorides

	BeBeCl4		BeMgCl4	BeBeCl4		BeMgCl4
Electrostatic	-355.5		-343.8	30.3		35.0
Exchange	-322.0		-249.4	27.5		25.4
Repulsion	900.9		705.7			
Polarization	-386.9		-292.8	33.0		29.8
Dispersion	-107.2		-95.0	9.1		9.7
Total E	-270.5		-275.3			

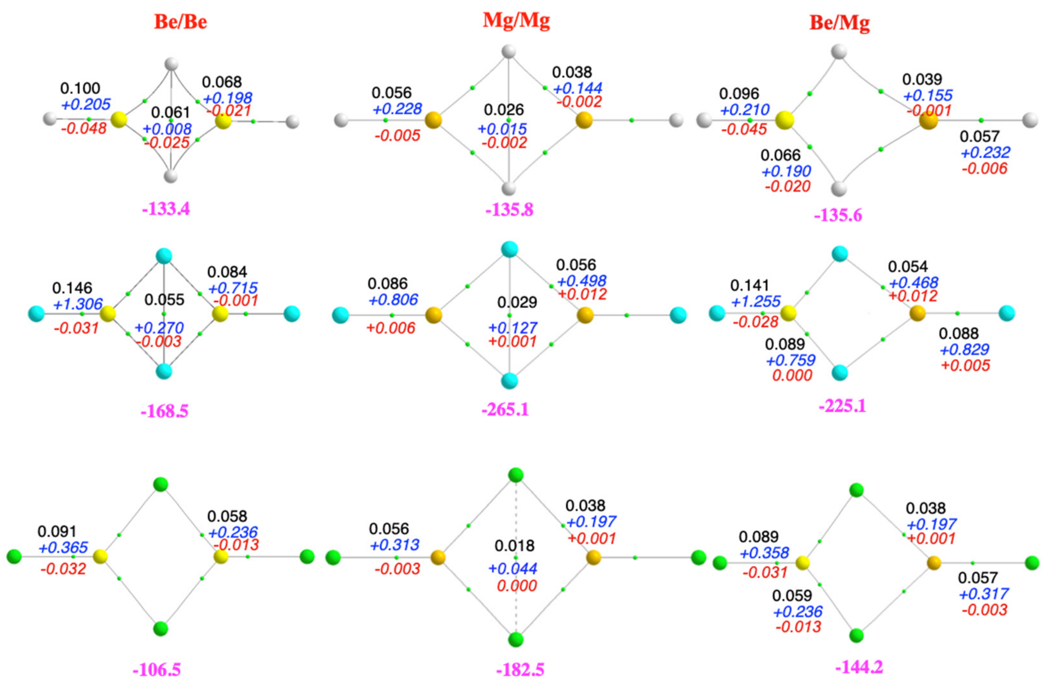

Figure S3. Molecular graphs of the homo and heterodimers involving BeX_2 and MgX_2 (X = H, F, Cl) monomers. The electron density (black), its Laplacian (blue italic) and the energy density (red italic) at the bond critical points (BCPs) are in

a.u. The numbers in magenta are the stabilization enthalpies in kJ·mol^{-1}.

Table S2. AdNPD orbital list for the $BeBeX_4$, $MgMgX_4$ and $BeMgX_4$ dimers

Be_2H_4:

---- AdNDP orbital list ----
\#   1 Occ: 1.9918 Atom: 1Be   2H
\#   2 Occ: 1.9918 Atom: 4Be   5H
\#   3 Occ: 1.9642 Atom: 3H   4Be   6H
\#   4 Occ: 1.9642 Atom: 1Be   3H   6H
Total occupation number in above orbitals: 7.9119

Residual valence electrons of all atoms in the search list: 0.088505

Mg_2H_4:

---- AdNDP orbital list ----
\#   1 Occ: 1.9959 Atom: 1Mg   3H
\#   2 Occ: 1.9959 Atom: 4Mg   6H
\#   3 Occ: 1.9894 Atom: 1Mg   2H   5H
\#   4 Occ: 1.9894 Atom: 2H   4Mg   5H
Total occupation number in above orbitals: 7.9707

Residual valence electrons of all atoms in the search list: 0.030936

$BeMgH_4$:

---- AdNDP orbital list ----
\#   1 Occ: 1.9975 Atom: 4Mg   5H
\#   2 Occ: 1.9895 Atom: 1Be   3H
\#   3 Occ: 1.9925 Atom: 1Be   2H   6H
\#   4 Occ: 1.9562 Atom: 1Be   4Mg   6H
Total occupation number in above orbitals: 7.9357

Residual valence electrons of all atoms in the search list: 0.064916

Be_2F_4:

---- AdNDP orbital list ----
\#   1 Occ: 1.9964 Atom: 2F
\#   2 Occ: 1.9964 Atom: 5F
\#   3 Occ: 1.9951 Atom: 3F
\#   4 Occ: 1.9951 Atom: 6F
\#   5 Occ: 1.9909 Atom: 5F
\#   6 Occ: 1.9909 Atom: 2F
\#   7 Occ: 1.9867 Atom: 3F

8 Occ: 1.9867 Atom: 6F
9 Occ: 1.9993 Atom: 1Be 3F
10 Occ: 1.9993 Atom: 4Be 6F
11 Occ: 1.9992 Atom: 1Be 3F
12 Occ: 1.9992 Atom: 4Be 6F
13 Occ: 1.9986 Atom: 1Be 2F
14 Occ: 1.9986 Atom: 1Be 5F
15 Occ: 1.9986 Atom: 4Be 5F
16 Occ: 1.9986 Atom: 2F 4Be
Total occupation number in above orbitals: 31.9295

Residual valence electrons of all atoms in the search list: 0.070317

Mg_2F_4

---- AdNDP orbital list ----
1 Occ: 1.9992 Atom: 3F
2 Occ: 1.9992 Atom: 5F
3 Occ: 1.9980 Atom: 2F
4 Occ: 1.9980 Atom: 6F
5 Occ: 1.9964 Atom: 2F
6 Occ: 1.9964 Atom: 6F
7 Occ: 1.9949 Atom: 3F
8 Occ: 1.9949 Atom: 5F
9 Occ: 1.9944 Atom: 3F
10 Occ: 1.9944 Atom: 5F
11 Occ: 1.9991 Atom: 1Mg 3F
12 Occ: 1.9991 Atom: 4Mg 5F
13 Occ: 1.9989 Atom: 2F 4Mg
14 Occ: 1.9989 Atom: 1Mg 6F
15 Occ: 1.9989 Atom: 1Mg 2F
16 Occ: 1.9989 Atom: 4Mg 6F
Total occupation number in above orbitals: 31.9600

Residual valence electrons of all atoms in the search list: 0.040214

$BeMgF_4$

---- AdNDP orbital list ----
1 Occ: 1.9992 Atom: 6F
2 Occ: 1.9962 Atom: 5F
3 Occ: 1.9962 Atom: 2F
4 Occ: 1.9952 Atom: 3F
5 Occ: 1.9946 Atom: 6F
6 Occ: 1.9942 Atom: 6F

\# 7 Occ: 1.9919 Atom: 5F
\# 8 Occ: 1.9919 Atom: 2F
\# 9 Occ: 1.9998 Atom: 1Be 3F
\# 10 Occ: 1.9996 Atom: 1Be 3F
\# 11 Occ: 1.9993 Atom: 1Be 3F
\# 12 Occ: 1.9991 Atom: 4Mg 6F
\# 13 Occ: 1.9989 Atom: 1Be 2F
\# 14 Occ: 1.9989 Atom: 1Be 5F
\# 15 Occ: 1.9974 Atom: 2F 4Mg
\# 16 Occ: 1.9974 Atom: 4Mg 5F
Total occupation number in above orbitals: 31.9498

Residual valence electrons of all atoms in the search list: 0.049794

Be_2Cl_4

---- AdNDP orbital list ----
\# 1 Occ: 1.9950 Atom: 2Cl
\# 2 Occ: 1.9950 Atom: 5Cl
\# 3 Occ: 1.9945 Atom: 3Cl
\# 4 Occ: 1.9945 Atom: 6Cl
\# 5 Occ: 1.9859 Atom: 5Cl
\# 6 Occ: 1.9859 Atom: 2Cl
\# 7 Occ: 1.9835 Atom: 3Cl
\# 8 Occ: 1.9835 Atom: 6Cl
\# 9 Occ: 1.9995 Atom: 1Be 2Cl
\# 10 Occ: 1.9995 Atom: 4Be 5Cl
\# 11 Occ: 1.9984 Atom: 1Be 2Cl
\# 12 Occ: 1.9984 Atom: 4Be 5Cl
\# 13 Occ: 1.9973 Atom: 1Be 6Cl
\# 14 Occ: 1.9973 Atom: 1Be 3Cl
\# 15 Occ: 1.9973 Atom: 3Cl 4Be
\# 16 Occ: 1.9973 Atom: 4Be 6Cl
Total occupation number in above orbitals: 31.9028

Residual valence electrons of all atoms in the search list: 0.096778

Mg_2Cl_4

---- AdNDP orbital list ----
\# 1 Occ: 1.9987 Atom: 3Cl
\# 2 Occ: 1.9987 Atom: 6Cl
\# 3 Occ: 1.9983 Atom: 5Cl

4 Occ: 1.9983 Atom: 2Cl
5 Occ: 1.9943 Atom: 3Cl
6 Occ: 1.9943 Atom: 6Cl
7 Occ: 1.9880 Atom: 2Cl
8 Occ: 1.9880 Atom: 5Cl
9 Occ: 1.9856 Atom: 2Cl
10 Occ: 1.9856 Atom: 5Cl
11 Occ: 1.9995 Atom: 1Mg   2Cl
12 Occ: 1.9995 Atom: 4Mg   5Cl
13 Occ: 1.9989 Atom: 1Mg   6Cl
14 Occ: 1.9989 Atom: 1Mg   3Cl
15 Occ: 1.9989 Atom: 4Mg   6Cl
16 Occ: 1.9989 Atom: 3Cl   4Mg
Total occupation number in above orbitals: 31.9243

Residual valence electrons of all atoms in the search list: 0.075740

$BeMgCl_4$

---- AdNDP orbital list ----
1 Occ: 1.9983 Atom: 5Cl
2 Occ: 1.9967 Atom: 3Cl
3 Occ: 1.9967 Atom: 6Cl
4 Occ: 1.9948 Atom: 2Cl
5 Occ: 1.9888 Atom: 3Cl
6 Occ: 1.9888 Atom: 6Cl
7 Occ: 1.9874 Atom: 5Cl
8 Occ: 1.9871 Atom: 2Cl
9 Occ: 1.9996 Atom: 4Mg   5Cl
10 Occ: 1.9994 Atom: 4Mg   5Cl
11 Occ: 1.9994 Atom: 1Be   2Cl
12 Occ: 1.9987 Atom: 1Be   3Cl
13 Occ: 1.9987 Atom: 1Be   6Cl
14 Occ: 1.9985 Atom: 1Be   2Cl
15 Occ: 1.9976 Atom: 3Cl   4Mg
16 Occ: 1.9976 Atom: 4Mg   6Cl
Total occupation number in above orbitals: 31.9282

Residual valence electrons of all atoms in the search list: 0.071941

Table S3. Wiberg bond indexes for Be_2X_4, Mg_2X_4, $BeMgX_4$ (X = H, F, Cl)

Cluster	Bonds	Wiberg indexes
Be_2H_4	Be-H	0.600 / 0.228
Mg_2H_4	Mg-H	0.518 / 0.154
$BeMgH_4$	Be-H<br>Mg-H	0.570 / 0.274<br>0.536 / 0.126
Be_2F_4	Be-F	0.256 / 0.125
Mg_2F_4	Mg-F	0.109 / 0.055
$BeMgF_4$	Be-F<br>Mg-F	0.242 / 0.146<br>0.111 / 0.052
Be_2Cl_4	Be-Cl	0.445 / 0.268
Mg_2Cl_4	Mg-Cl	0.315 / 0.152
$BeMgCl_4$	Be-Cl<br>Mg-Cl	0.434 / 0.300<br>0.320 / 0.148

Table S4. Interatomic distances in the cycles **A** of Be_2X_4 and Mg_2X_4 (X = H, Cl). All values are in Å. The atoms numbering is shown in the attached figure.

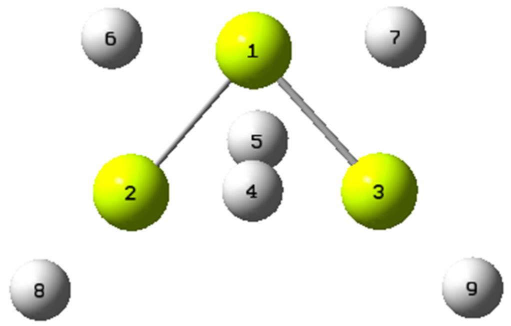

Be_2X_4 and Mg_2X_4 / Cycle **A**		
**X = H**		
Atoms involved	**1-2** and **1-3**	**2-3**
Be-Be distances	1.860	2.416
Mg-Mg distances	2.603	3.124
Atoms involved	**1-6** and **1-7** / **2-6** and **3-7**	**2-4** and **3-4** / **2-8** and **3-9**

Be-H distances	1.386 / 1.553	1.581 / 1.320
Mg-H distances	1.756/ 1.984	1.988 / 1.687
Atoms involved	4-5	
H-H (in Be cycle)	1.972	
H-H (in Mg cycle)	2.295	
**X = Cl**		
Atoms involved	**1-2** and **1-3**	**2-3**
Be-Be distances	2.280	3.167
Mg-Mg distances	2.924	3.739

Table S5. LMO-EDA analysis for the $BeBeBeF_6$ trimers (all values in kJ·mol^{-1})

	Energies (kJ·mol^{-1}) of the different components			% of the attractive components to the total E		
	Cycle **A**	Cycle **B**	linear	Cycle **A**	Cycle **B**	linear
Electrostatic	-993.4	-873.7	-991.6	39.7	40.5	41.9
Exchange	-652.8	-470.5	-540.3	26.1	21.8	22.8
Repulsion	**1934.2**	**1468.7**	**1672.5**			
Polarization	-608.6	-604.3	-638.3	24.3	28.0	27.0
Dispersion	-250.6	-208.3	-196.5	10.0	9.7	8.3
Total E	-571.1	-688.0	-694.2			

Table S6. Relative stabilities (kJ·mol^{-1}) for the MgF_2 trimers obtained by different theoretical approaches.

	**GA/DFT**[a]	**M06-2X**	**G4**
Linear	0.0	0.0	0.0
Hexagonal cycle	40.2	56.9	54.1
cycle	32.2	20.7	29.4
cycle	43.1	13.8	25.4

Table S7. MBIE analysis of linear heterotrimer complexes formed by BeX_2 and MgX_2 (X = H, F, Cl). All values in kJ·mol^{-1}.

Ternary complex	$E_R(A)$	$E_R(B)$	$E_R(C)$	$D^2E(AB)$	$D^2E(AC)$	$D^2E(BC)$	$D^3E(ABC)$	E_{total}
$BeBeMgH_6$	58.5	110.2	32.7	-262.8	8.6	-247.0	-33.2	-333.0
$BeMgBeH_6$	67.4	57.2	67.4	-240.5	-0.3	-240.5	-4.0	-293.2
$BeMgMgH_6$	67.2	62.0	37.8	-241.6	-0.3	-214.6	-8.3	-297.8
$MgBeMgH_6$	31.6	117.5	31.6	-247.1	11.2	-247.3	-31.5	-333.8
$BeBeMgF_6$	82.6	172.4	42.6	-347.7	-1.9	-364.7	0.4	-416.4
$BeMgBeF_6$	86.6	80.7	86.6	-358.0	-2.4	-358.0	0.5	-464.1
$BeMgMgF_6$	86.2	81.5	43.1	-359.8	-2.6	-355.2	0.5	-506.3
$MgBeMgF_6$	40.9	176.1	40.9	-365.3	0.6	-365.3	4.8	-467.2
$BeBeMgCl_6$	80.3	168.3	39.7	-276.8	-0.5	-2821	7.9	-263.1
$BeMgBeCl_6$	88.6	77.0	88.6	-276.9	-1.9	-276.9	0.4	-301.1
$BeMgMgCl_6$	88.5	81.2	44.5	-277.5	-2.5	-276.0	1.3	-340.4
$MgBeMgCl_6$	39.3	175.9	39.3	-282.1	-1.0	-282.1	10.8	-299.7

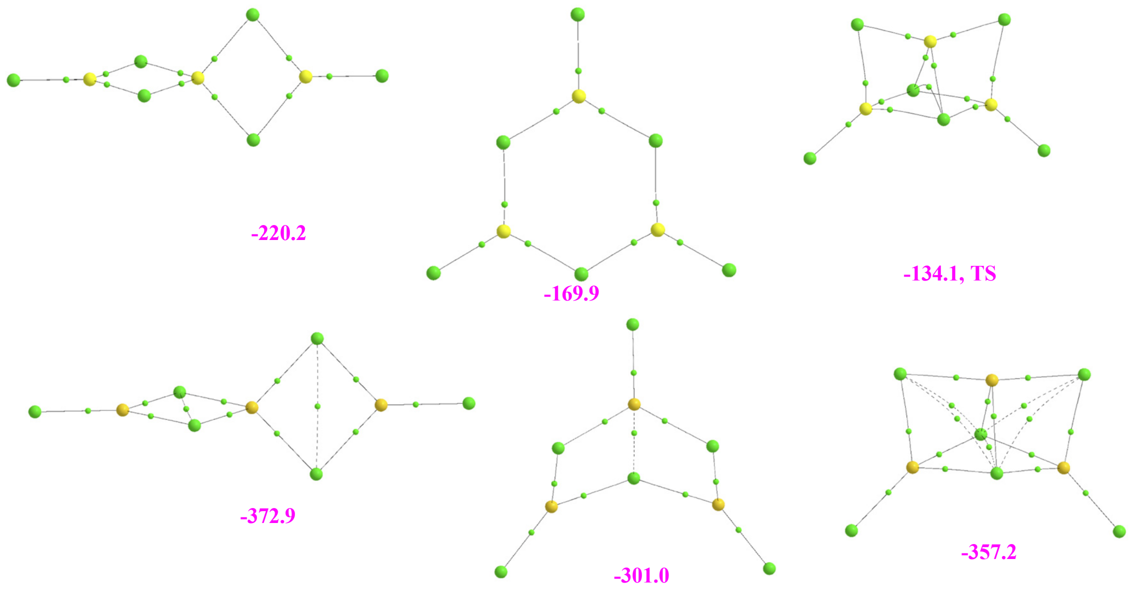

Figure S4. Bond paths and stabilization enthalpies (kJ·mol^{-1}) for the $BeCl_2$ and $MgCl_2$ homotrimers.

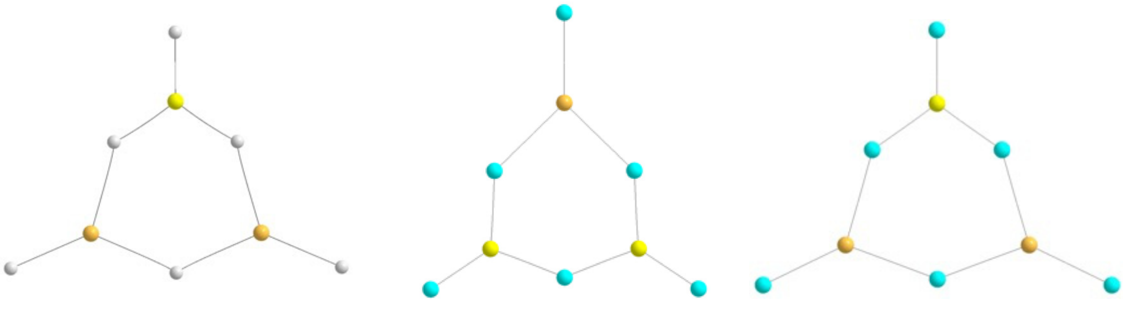

-243.2	-397.8	-442.9
68.1	57.1	54.7

Figure S5. Bond paths for the stable hexagonal heterotrimers $BeCl_2$ and $MgCl_2$ homotrimers, showing their stabilization (black) and relative enthalpies (blue) with respect to the corresponding linear heterotrimer in kJ·mol^{-1}.

Table S8. MBIE analysis of $BeBeMgH_6$ and $BeMgMgH_6$ heterotrimer complexes. All values in kJ·mol^{-1}.

$BeBeMgH_6$								
Ternary complex	E_R(A)	E_R(B)	E_R(C)	Δ^2E(AB)	Δ^2E(AC)	Δ^2E(BC)	Δ^3E(ABC)	E_{total}
linear	58.5	110.2	32.7	-262.8	8.6	-247.0	-33.2	-333.0
Cycle **E**	18.1	89.47	54.4	-143.41	-115.49	-182.33	-59.1	-338.36
$BeMgMgH_6$								
Ternary complex								
linear	67.2	62.0	37.8	-241.6	-0.3	-214.6	-8.3	-297.8
Cycle **C**	36.5	60.2	60.2	-152.8	-152.7	-147.5	-45.8	-341.9

Table S9. Molecular graphs of cycles **C-G** of $BeX_2BX_2MgX_2$ and $BeX_2MgX_2Mgx_2$ (X = F, Cl) and their stabilization enthalpies (bold numbers). In blue their relative stabilities with respect to the corresponding linear trimer are also given. All values in kJ·mol^{-1}.

Fluorides	BeBeMg		BeMgMg	
**C**	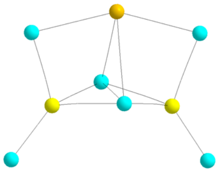	**-342.5**<br>112.4	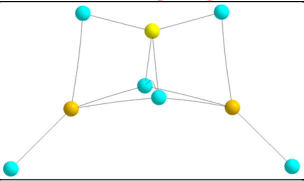	**-426.6**<br>71.0
**D**	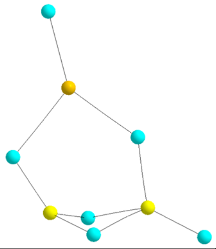	**-360.6**<br>94.3	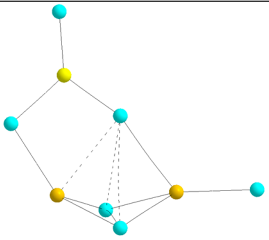	**-466.0**<br>31.6
**E**	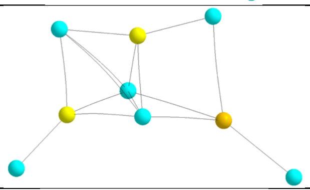	**TS**	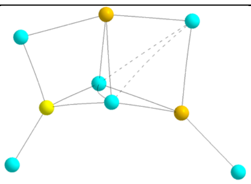	**-437.3**<br>60.3
**F**	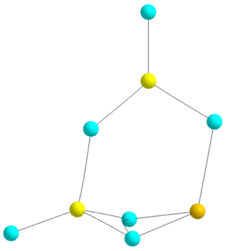	**-372.4**<br>82.5	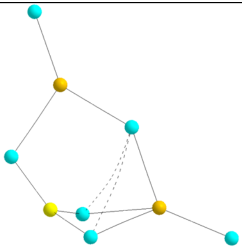	**-455.0**<br>42.6
**G**	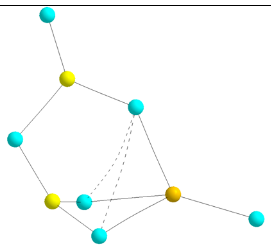	**-398.4**<br>56.5	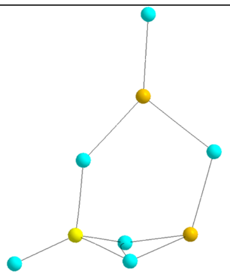	**-423.0**<br>74.6
Chlorides				
**C**	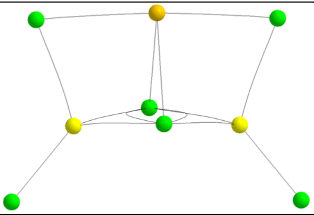	**-197.1**<br>97.5	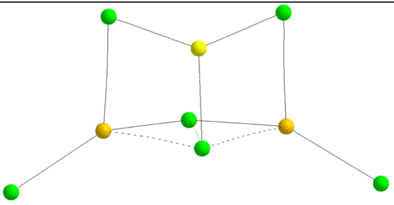	**-283.3**<br>50.5
**D**		**TS**	**Does not exist**[a]	

**E**	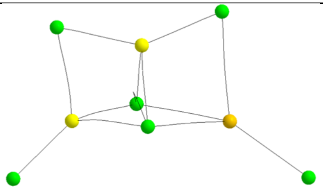	**-207.7** 86.9	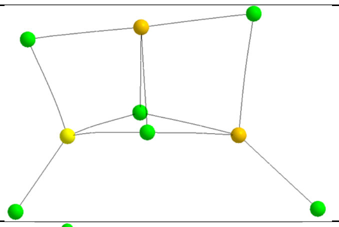	**-277.5** 56.3
**F**	**Does not exist**[a]		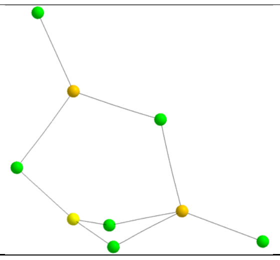	**-289.8**
**G**	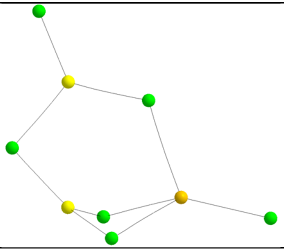	**-241.0** 53.6	**Does not exist**[a]	44.0

[a] These structures are not stable as they collapse to the global minimum.

Table S10. MBIE analysis of heterotrimer complexes for fluorides and chlorides. All values in kJ·mol^{-1}.

Ternary complex	E_R(A)	E_R(B)	E_R(C)	Δ^2E(AB)	Δ^2E(AC)	Δ^2E(BC)	Δ^3E(ABC)	E_{total}
$BeBeMgF_6$_linear	82.6	172.4	42.6	-347.7	-1.9	-364.7	0.4	-416.4
$BeMgBeF_6$_linear	86.6	80.7	86.6	-358.0	-2.4	-358.0	0.5	-464.1
$BeMgMgF_6$_cyc_ hexagonal	94.2	44.6	50.5	-139.2	-224.0	-195.8	-81.6	-451.4
$BeBeMgF_6$ cycle **E**	39.5	171.0	86.6	-184.4	-171.1	-353.9	83.0	-329.2
$BeBeMgF_6$ cycle **C**	27.8	176.7	176.6	-209.9	-209.5	-367.9	57.3	-348.9
$BeMgMgF_6$_linear	86.2	81.5	43.1	-359.8	-2.6	-355.2	0.5	-506.3
$MgBeMgF_6$_linear	40.9	176.1	40.9	-365.3	0.6	-365.3	4.8	-467.2
BeMgMgF6_cyc_ hexagonal	94.2	44.6	50.48	-139.2	-224.0	-195.8	-81.6	-451.4
BeMgMgF6 cycle **E**	21.9	182.5	84.8	-232.4	-188.5	-367.2	54.1	-444.6
BeMgMgF6 cycle **C**	51.1	92.6	92.4	-207.2	-207.2	-336.4	80.9	-433.9
BeBeMgCl6_linear	80.3	168.3	39.7	-276.8	-0.5	-282.1	7.9	-263.1
BeMgBeCl6_linear	88.6	77.0	88.6	-276.9	-1.9	-276.9	0.4	-301.1
BeBeMgCl6_cyc_ hexagonal_NP	126.6	63.7	62.1	-89.9	-291.3	-63.7	-31.4	-223.9
BeBeMgCl6 cycle **E**	54.2	151.4	73.0	-167.1	-149.0	-264.9	90.0	-212.3
BeBeMgCl6 cycle **C**	14.0	158.2	157.9	-162.7	-162.5	-278.2	72.0	-201.4
BeMgMgCl6_linear	88.5	81.2	44.5	-277.5	-2.57	-276.0	1.3	-340.4
MgBeMgCl6_linear	39.3	175.9	39.35	-282.1	-1.0	-282.1	10.8	-299.7
BeBeMgCl6_cyc_ hexagonal_NP	133.7	48.3	87.7	-116.7	-292.0	-117.2	-50.0	-306.2
BeMgMgCl6 cycle **E**	14.9	166.5	77.5	-182.7	-149.3	-275.6	66.0	-282.7
BeMgMgCl6 cycle **C**	65.6	76.9	76.9	-168.5	-168.5	-258.1	86.8	-288.8
